# Association Between High Serum Matrix Metalloproteinase-9 and MMP-9 (-1562C>T) Polymorphism in Patients With ST-Elevation Acute Myocardial Infarction

**DOI:** 10.4021/cr210w

**Published:** 2012-09-20

**Authors:** Budi Y. Setianto, Sofia Mubarika, Bambang Irawan, Indwiani Astuti

**Affiliations:** a Department of Cardiology and Vascular Medicine, Faculty of Medicine Gadjah Mada University - Dr. Sardjito Hospital, Yogyakarta, Indonesia; b Department of Histology and Molecular Biology, Faculty of Medicine Gadjah Mada University, Yogyakarta, Indonesia; c Department of Pharmacology, Faculty of Medicine Gadjah Mada University, Yogyakarta, Indonesia

**Keywords:** MMP-9, Polymorphism: Acute coronary syndrome, STEMI

## Abstract

**Background:**

Matrix metalloproteinase (MMP)-9 is excessively expressed in frail region of atherosclerotic plaque and released in circulation following plaque rupture. High MMP-9 level associated with severity of occluded thrombus and subsequent myocardial infarction. MMP-9 (-1562C>T) polymorphism associated with acute myocardial infarction, however conflicting data present regarding impact of MMP-9 (-1562C>T) polymorphism on circulating MMP-9 level in acute myocardial infarction with ST-elevation (STEMI), clinical entity represents totally occluded coronary thrombus.

**Methods:**

We enrolled consecutively subjects with acute coronary syndrome treated in intensive coronary care unit. Acute coronary syndrome diagnosis were classified into STEMI and non-ST-elevation acute coronary syndrome (NSTEACS). Seventy consecutive subjects were enrolled for this study, 31 subjects with STEMI and 39 subjects with NSTEACS.

**Results:**

On admission serum MMP-9 level, measured with sandwich enzyme immunoassay, were higher in STEMI as compared with NSTEACS (1,574.2 ± 604.1 ng/mL vs. 1,104.4 ± 591.5 ng/mL, P < 0.01). Proportion of subjects with MMP-9 (-1562C>T) polymorphism, analyzed with PCR-RFLP, were higher in STEMI as compared with NSTEACS (66.7% vs. 33.3%, P = 0.15). T allele frequency was almost twice in STEMI as compared to in NSTEACS. Almost all (83%) subjects with MMP-9 (-1562C>T) polymorphism had high serum MMP-9 level (> 1,334.5 ng/mL) during STEMI, whereas in NSTEACS all subjects had low level.

**Conclusion:**

MMP-9 (-1562C>T) polymorphism associated with high serum MMP-9 level in patients with STEMI.

## Introduction

Acute coronary syndrome progression involves rupture or erosion of coronary atherosclerotic plaque and ensuing coronary luminal thrombus formation [1]. Rupture of plaque is attributed to damage of intimal collagen and degradation of extracellular matrix by proteinase [2, 3]. Among proteinase, matrix metalloproteinase (MMP)-9 is abundantly expressed in frail region of coronary plaque [2, 3].

MMP is an endoproteinase with the ability to degrade the extracellular matrix in a variety of tissue [4]. Four groups of MMP family have been recognized based on their substrate specificity and major structure, including MMP-9, which is included in group 2 metalloproteinase or gelatinases [4]. Rupture-prone area of atherosclerotic plaque excessively expressed MMP-9, which becoming the source of elevated serum MMP-9 during plaque rupture [5].

Single nucleotide polymorphism (SNP) in the MMP-9 gene promoter, namely the transition of C (cytosine) by T (thymidine) at position -1562, is known to be risk factor for acute myocardial infarction [6, 7]. The frequency of MMP-9 (-1562C>T) SNP in acute myocardial infarction is higher than controls and related with the complexity of coronary lesion [6, 8, 9]. However, conflicting data still present regarding the impact of MMP-9 (-1562C>T) polymorphism on serum MMP-9 level in patients with STEMI and NSTEACS.

The purpose of this study was to investigate: 1) the difference of serum MMP-9 level in STEMI compared to NSTEACS; 2) the difference of the frequency of MMP-9 (-1562C>T) polymorphisms in STEMI compared to NSTEACS and 3) the association between serum MMP-9 level with MMP-9 (-1562C>T) polymorphism in STEMI compared to NSTEACS.

## Methods

### Subjects

The subjects of this study were patients with acute coronary syndromes hospitalized in intensive coronary care unit of Dr. Sardjito Hospital, Yogyakarta, Indonesia. These subjects were enrolled consecutively during periods of June 2009 and Augusts 2010. Acute coronary syndromes were diagnosed based on criterions released by Indonesian Association of Cardiovascular Specialists (PERKI) [10] and divided into two clinical groups, namely, ST-elevation acute myocardial infarction (STEMI) and non-ST-elevation acute coronary syndromes (NSTEACS). The criterions for STEMI are clinical ischemic symptoms and electrocardiographic sign of ST-segment elevation or new left bundle branch block (LBBB). The criterions for NSTEACS are clinical ischemic symptoms and electrocardiographic signs of ischemic but without ST-segment elevation. Inclusion criterions for this study were patients over 18 years of age, patient had onset of ischemic symptoms less than 24 hour prior to hospital arrival and patients agreed to participate in the study by signing informed consent. Exclusion criterions from this study were patients with comorbidies, namely, end-stage chronic renal failure, chronic congestive heart failure, acute stroke, acute infections, chronic inflammatory diseases, venous thromboembolism, malignancy, and hematological disorders. Committee of Ethics Faculty of Medicine Universitas Gadjah Mada, Yogyakarta, Indonesia, approved the study.

### Serum MMP-9 examination

Peripheral venous blood was withdrawn on admission before thrombolysis or coronary intervention therapy for subjects with STEMI. For MMP-9 measurement, a 5 mL peripheral venous blood was collected in vacutainer and allowed to clot for 30 minutes, the sample was subsequently centrifuged at 1,000 rpm for 15 min and supernatant was stored at -80 °C until assayed. MMP-9 levels were measured by sandwich enzyme immunoassay methods with a Quantikine Human MMP-9 Immunoassay kit (R&D Systems Inc., Minneapolis, Minneapolis, USA).

### MMP-9 (-1562C>T) polymorphism examination

For MMP-9 (-1562C>T) polymorphism analysis, a 5 mL peripheral venous blood was obtained and put into EDTA-containing tubes. Total DNA was isolated manually from blood leukocytes using guanidine isothiocyanate method. Primer sequences used were: forward primer 5’-GCCTGGCACATAGTAGGCCC-3' and reverse primer 5’-CTTCCTAGCCAGCCGGCATC-3’. SNP polymorphisms of MMP-9 (-1562C>T) were identified by DNA sequencing using a PCR-RFLP (Restriction Fragment Length Polymorphism). Briefly, the reaction was performed in a 24 µL PCR mixture (10 × PCR buffer 2.5 µL, dNTP mix. 25mM 0.2 µL, Taq polymerase 0.2 µL, forward primer (1.59 µg/µL) 1 µL, reverse primer (2.01 µg/µl) 1 µl and steril aquadest 19.1 µL) and 1 µL total DNA. PCR condition was as follows: heating at 95 °C 5 min, denaturation 95 °C 50 min, annealing 63 °C 1 h 5 min and synthesis 72 °C 1 h 5 min. PCR products were run on 2% agarose gel and visualized with ethidium bromide. Band identified then cut and purified with PureLinkTM Quick Gel Extraction Kit (Invitrogen, Carlsbad, California, USA) according to manufacturer manual. Squencing was performed with DYEnamic ET Terminator Cycle Sequencing Kit (GELifeScience, Buckinghamshire, UK) and the squencing condition was as follows: 95 °C 20 sec, 50 °C sec and 60 °C 1 min for 40 cycles. Squencing product was then analyzed on MegaBACE DNA Analysis Systems. Nucleotide squence from each sample was analyzed with Bioedit software (Carlsbad, Californis, USA).

### Routine laboratory examination

For hematology examination, blood sample was analyzed with Coulter HmX Hematology Analyzer (Beckman Coulter, USA) and for blood chemistry examination, blood sample was analyzed with Synchron Automatic Chemistry Analyzer (Beckman Coulter, USA). These procedures were conducted in the hospital laboratory, Dr. Sardjito Hospital, Yogyakarta, Indonesia.

### Statistical analysis

Comparison of continues variables was analyzed with unpaired Student’s-t test. Comparison of categorical variables was analyzed with chi-squared tests or Fisher exact test. Independent association for multiple variables was analyzed with multiple logistic regression analysis. Receiver operator characteristic (ROC) curve was constructed to determined the best cut-off value of MMP-9 to predict STEMI. Prevalence risk of high MMP-9 in STEMI was calculated based on cut-off value. Extrapolation of the genetic population of the research was confirmed with Hardy Weinberg equilibrium. Statistic analysis was performed with SPSS version 13.0 (SPSS, Chicago, USA). Statistical significance was considered when P < 0.05.

## Results

### Characteristics of subjects

During the study period, we enrolled 80 subjects, however as many as 10 subjects were excluded from analysis due to incomplete data. Among 70 subjects, STEMI was diagnosed in 31 subjects and NSTEACS in 39 subjects. Characteristics of the subjects were shown in Table 1. Demography data showed no significant different between groups. Clinical presentation showed STEMI group had approximately 4 hour shorter onset of ischemic sign before reaching hospital than in NSTEACS group (P = 0.02). Killip class on admission was comparable between group. Haematology examination showed higher leucocyte count in STEMI groups as compared with NSTEACS (P < 0.001), whereas haemoglobin level and platelet count were equal. In blood chemistry measurement, LDL cholesterol level was significantly higher in STEMI group (P = 0.01), triglyceride level was higher in NSTEACS, however this difference did not reach statistical significance (P = 0.07).

**Table 1:** Characteristics of Subjects Divided by Subjects With STEMI and NSTEACS

Variables	STEMI (n = 31)	NSTEACS (n = 39)	P value
Demography			
Gender, n (%)			0.09
Male	28 (49.1%)	29 (50.9%)	
Female	3 (23.1%)	10 (76.9%)	
Age (years), mean ± SD	56.2 ± 11.3	60.4 ± 9.9	0.10
Risk factors, n (%)			
Diabetes mellitus	8 (40%)	12 (60%)	0.65
Hypertension	17 (38.6%)	27 (61.4%)	0.22
Current smoking	20 (45.5%)	24 (54.5%)	0.79
Dyslipidemia	18(38.3%)	29 (61.7%)	0.15
Clinical Presentation			
Onset (h), mean ± SD	5.9 ± 5.8	9.6 ± 7.6	0.02
Killip class, n (%)			0.84
Killip class I	26 (44.8%)	32 (55.2%)	
Killip class II-IV	5 (41.7%)	7 (58.3%)	
Laboratory Results			
Haematology, mean ± SD			
Haemoglobin (g/dL)	13.5 ± 2.2	13.6 ± 1.8	0.98
Leucocyte count (10^3^/mm^3^)	13.8 ± 4.6	10.0 ± 3.3	< 0.001
Platelet count (10^3^/mm^3^)	278.3 ± 72.9	251.5 ± 75.0	0.14
Chemistry, mean ± SD			
Creatinine (mg/dL)	1.4 ± 0.6	1.6 ± 1.6	0.57
Glucose (mg/dL)	173.5 ± 100.4	150.6 ± 65.7	0.13
Total cholesterol (mg/dL)	202.3 ± 49.2	181.8 ± 53.4	0.10
LDL cholesterol (mg/dL)	135.1 ± 44.9	109.6 ± 38.8	0.01
HDL cholesterol (mg/dL)	40.6 ± 9.0	36.5 ± 13.5	0.15
Triglyceride (mg/dL)	117.2 ± 54.9	162.3 ± 115.8	0.07

### Elevated serum MMP-9 was independently associated with STEMI

**Figure 1:**
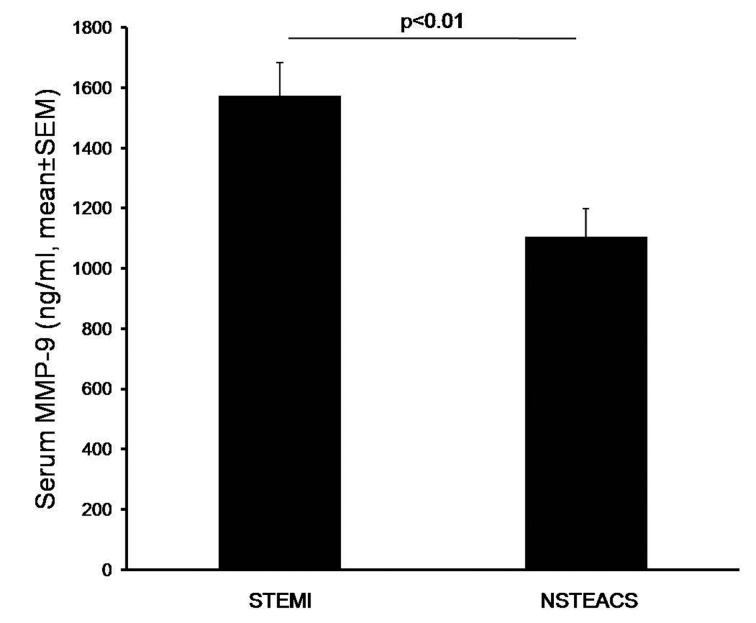
Serum MMP-9 level was significantly higher in subjects with STEMI than those in subjects with NSTEACS.

Serum MMP-9 level was significantly higher in subjects with STEMI as compared to that in those with NSTEACS (1,574.2 ± 604.1 ng/mL versus 1,104.4 ± 591.5 ng/mL, P = 0.005, respectively) (Fig. 1).

To determine whether serum MMP-9 independently associated with STEMI, we performed multivariable logistic regression analysis that included all variables, namely, demography, clinical presentation and laboratory results. This analysis showed that serum MMP-9 independently associated with STEMI (adjusted OR 1.002; 95%CI 1.001 - 1.003, P = 0.003). Other independent variables were hour of onset (adjusted OR 0.891; 95%CI 0.796 - 0.996, P = 0.043) and leucocyte count (adjusted OR 1.311; 95%CI 1.110 - 1.550, P = 0.003) (Table 2).

**Table 2:** Independent Variables Associated With STEMI

Variables	β	Adjusted OR	95%CI OR	P value
Onset (hour)	-0.116	0.891	0.796 - 0.996	0.043
Leucocyte count	0.271	1.311	1.110 - 1.550	0.003
MMP-9 level	0.002	1.002	1.001 - 1.003	0.003

**Figure 2:**
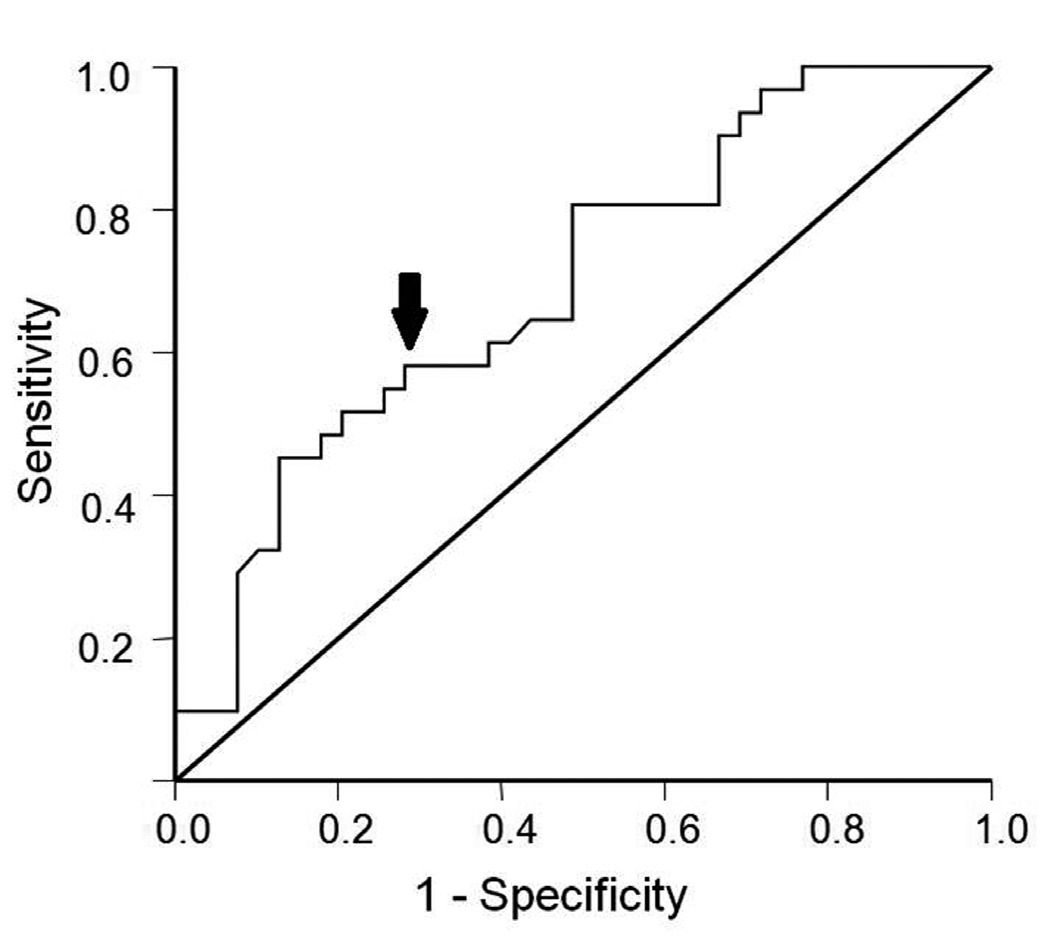
Receiver operator characteristic curve (ROC) of serum MMP-9 level to predict STEMI. Trade-off between sensitivity and specificity was best characterized at 1,334.5 ng/mL (arrow), which gave a sensitivity of 58.1% and specificity of 71.8%.

For further analysis, we determined the cut-off value of serum MMP-9 in our study to predict STEMI with receiver operator characteristic (ROC) curve analysis. ROC curve showed the best trade-off value of 1,334.5 ng/mL gave sensitivity of 58.1% and specificity of 71.8% (Fig. 2). Based on this cut-off value, we divided subjects into high MMP-9 (> 1,334.5 ng/mL) group and low MMP-9 (≤ 1334.5 ng/mL) group. Table 3 showed that prevalence ratio of high MMP-9 in subjects with STEMI was almost twice higher than that in subjects with NSTEACS (PR 1.96; 95%CI 1.30 - 9.50, P = 0.012).

**Table 3:** High Serum MMP-9 Level and Prevalence Ratio in STEMI

MMP-9 level (ng/mL)	STEMI (n = 31)	NSTEACS (n = 39)	Prevalence Ratio (PR) (95%CI)	P value
High MMP-9 ( >1,334.5)	18 (62.1%)	11 (37.9%)	1.96 (1.30 - 9.50)	0.012
Low MMP-9 (≤ 1,334.5)	13 (31.7%)	28 (68.3%)		

### MMP-9 (-1562C>T) polymorphism frequency was higher in STEMI

Total of nine patients had MMP-9 (-1562C>T) polymorphism, among them 6 (66.7%) patients belonged to STEMI and 3 (33.3%) belonged to NSTEACS. However this difference did not reach statistical significance (P = 0.150).

Table 4 showed the distribution of genotypes from MMP-9 (-1562C>T) polymorphism in STEMI and NSTEACS. In this study, 6 out of 31 subjects with STEMI (19.4%) had heterozygote C>T genotype whereas in NSTEACS, 2 out of 39 (5.1%). Homozygote C>C genotype was found in 25 (80.6%) in subjects with STEMI and 36 (92.3%) in NSTEACS. One subject in NSTEACS had homozygote T>T genotype. T allele frequency was higher in patients with STEMI as compared to those with NSTEACS (9.7% versus 5.1%, respectively). In the calculation of genetic populations using Hardy Weinberg equilibrium, we obtained the genotype distribution was not significantly different (P = 0.250).

**Table 4:** The MMP-9 (-1562C>T) Polymorphism Frequencies Based on the Transition Alleles

MMP-9 (−1562C>T) polymorphism	STEMI (n = 31)	NSTEACS (n = 39)	Total (n = 70)
MMP-9 (-1562C>T) genotypes			
Heterozygote C>T, n (%)	6 (19.4%)	2 (5.1%)	8 (11.4%)
Homozygote C>C, n (%)	25 (80.6%)	36 ((92.3%)	61 (87.1%)
Homozygote T>T, n (%)	0 (0%)	1 (2.6%)	1 (1.4%)
MMP-9 (-1562C>T) alleles			
C alleles	56 (90.3%)	74 (94.9)	130 (92.9)
T alleles	6 (9.7%)	4 (5.1%)	10 (7.1)

### MMP-9 (-1562C>T) polymorphism was associated with high MMP-9 level in STEMI

**Figure 3:**
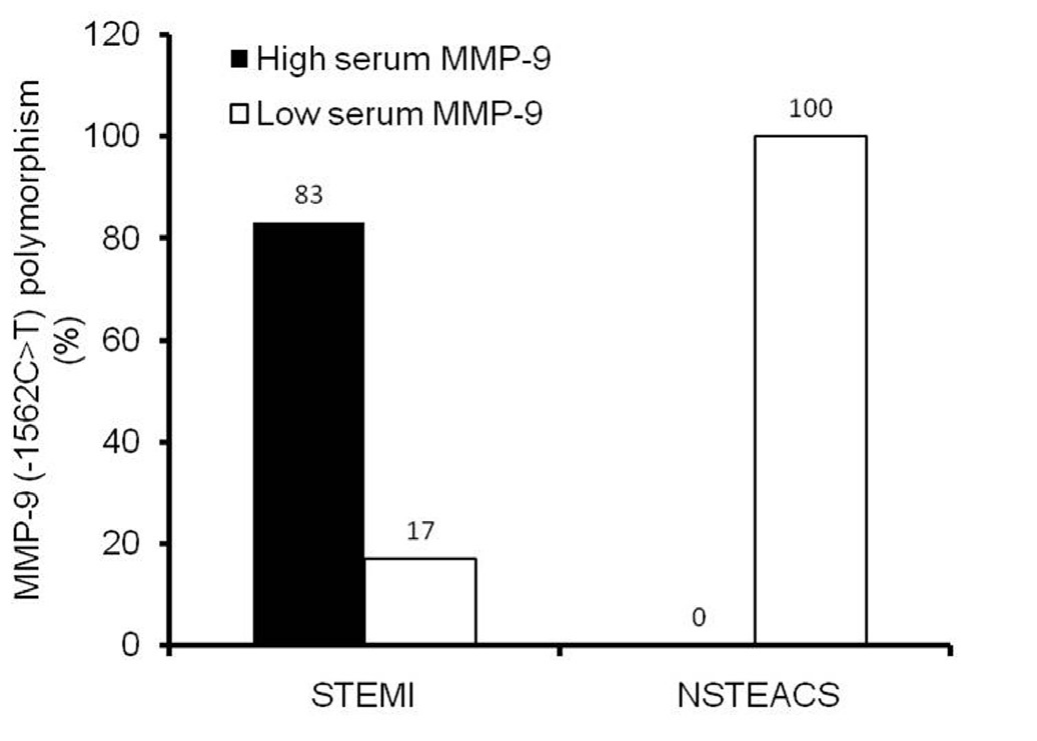
Proportion of MMP-9 (-1562C>T) polymorphism between subjects with STEMI and NSTEACS in association with serum MMP-9 level. MMP-9 (-1,562C>T) polymorphism associated with high serum MMP-9 (> 1334.5 ng/mL) level in STEMI subjects, inversely MMP-9 (-1,562C>T) polymorphism associated with low serum MMP-9 (≤ 1,334.5 ng/mL) in NSTEACS subjects (Fisher exact test: estimated risk 4, P = 0.048).

Based on serum MMP-9 cut-off value, we found that MMP-9 (-1562C>T) polymorphism was associated with high serum MMP-9 levels (> 1334.5 ng/mL) in subjects with STEMI (OR 4; 95%CI 0.733 - 21.838, P = 0.048). Five out of 6 subjects (83%) with MMP-9 polymorphism in STEMI group had high serum MMP-9 levels. Only 1 patient (17%) had low serum MMP-9 (≤ 1334.5 ng/mL). In NSTEACS group, all subjects with MMP-9 (-1562C>T) polymorphism had low serum MMP-9 (Fig. 3).

## Discussion

Our study provided two finding, firstly, high serum MMP-9 level measured in the early time of acute coronary syndrome was significantly elevated in patients with STEMI and secondly, MMP-9 (-1562C>T) polymorphism associated with high serum MMP-9 level in patients with STEMI.

Our study result showed that serum MMP-9 level was significantly increased in STEMI, indicated greater damage or degradation of extracellular matrix from plaque rupture. MMP-9 contributes to the vulnerability of coronary atherosclerotic plaques which tends to rupture or erode and precipitates acute coronary events [11]. Previous studies showed that plasma MMP-9 level was increased in patients with acute coronary syndromes and acute myocardial infarction [12, 13]. Elevated plasma MMP-9 level during acute myocardial infarction associated with subsequent ventricular remodeling [14].

MMP-9 (-1562C>T) polymorphism associated with higher plasma MMP-9 activity and contributed to the development of early onset coronary artery disease [15]. Fiotti et al [16] reported that the MMP-9 promoter microsatellite polymorphism associated with thin fibrous caps and large lipid core of coronary atherosclerosis. Study by Koh et al [8] found that MMP-9 (-1562C>T) polymorphism was significantly and independently associated with acute myocardial infarction. Furthermore, this study also suggested relation between MMP polymorphism with increased serum MMP-9 levels in acute myocardial infarction. Our study specify this finding in STEMI patients, who has total blockade of coronary artery due to plaque-driven occluded-thrombus. We found that patients with MMP-9 (-1562 C>T) polymorphism had increased serum MMP-9 during STEMI episodes. The estimated risk to develop increased serum MMP-9 levels were as much as 4 times. This may have consequences in the course of disease, especially in STEMI, which need further research.

Our study indicated heterozygote C>T was higher in STEMI subjects as compared to NSTEACS. Morgan et al [17] showed in -1562C>T polymorphism, -1562T allele had a higher transcriptional activity as compared to that of -1562C allele. Similarly, Kim et al [18] reported that the substitution of C>T at -1562 promoter will provide a higher promoter activity of the T-allelic promoter. This fact contributes to elevated MMP-9 in the plaque which is detected in serum during plaque rupture in subjects with MMP-9 (-1562C>T) polymorphism. We also found MMP-9 T allele frequency was higher in subjects with STEMI.

Despite several evidences of the role of MMP-9 polymorphism in coronary artery disease, some publications indicated conflicting conclusions regarding role of MMP-9 polymorphism in severity of coronary artery disease [9, 19, 20]. These similarities and differences in the results of previous studies possibly because of polymorphism that occurs in the promoter region will affect gene transcription and increase expression of MMP-9 [21]. Moreover in the activation of MMP-9 from pro-MMP, some interactions are still required such as TIMP, MT-MMP, plasmin, and feedback mechanism of MMP-9 itself [21].

In conclusions, serum MMP-9 level in acute coronary syndrome was significantly higher in STEMI than that in NSTEACS, whereas MMP-9 (-1562C>T) polymorphism has a trend to be higher in STEMI than that in NSTEACS. MMP-9 (-1562C>T) polymorphism was associated with high serum MMP-9 level in subject with STEMI.
